# Facile Preparation of CuS Nanoparticles from the Interfaces of Hydrophobic Ionic Liquids and Water

**DOI:** 10.3390/molecules24203776

**Published:** 2019-10-21

**Authors:** Yunchang Fan, Yingcun Li, Xiaojiang Han, Xiaojie Wu, Lina Zhang, Qiang Wang

**Affiliations:** 1 College of Chemistry and Chemical Engineering, Henan Polytechnic University, Jiaozuo 454003, China; fanyunchang@hpu.edu.cn (Y.F.); m13080151080@sohu.com (Y.L.); latiaowu@sina.com (X.W.); 2 Zhenhai District Center for Disease Control and Prevention, Ningbo 315200, China; zjsxhxj@163.com

**Keywords:** ionic liquids (ILs), copper sulfide (CuS), nanoparticles, photocatalytic activity

## Abstract

In this work, a two-phase system composed of hydrophobic ionic liquid (IL) and water phases was introduced to prepare copper sulfide (CuS) nanoparticles. It was found that CuS particles generated from the interfaces of carboxyl-functionalized IL and sodium sulfide (Na_2_S) aqueous solution were prone to aggregate into nanoplates and those produced from the interfaces of carboxyl-functionalized IL and thioacetamide (TAA) aqueous solution tended to aggregate into nanospheres. Both the CuS nanoplates and nanospheres exhibited a good absorption ability for ultraviolet and visible light. Furthermore, the CuS nanoplates and nanospheres showed highly efficient photocatalytic activity in degrading rhodamine B (RhB). Compared with the reported CuS nanostructures, the CuS nanoparticles prepared in this work could degrade RhB under natural sunlight irradiation. Finally, the production of CuS from the interfaces of hydrophobic IL and water phases had the advantages of mild reaction conditions and ease of operation.

## 1. Introduction

Copper sulfide (CuS), a *p*-type semiconductor, has attracted tremendous interest due to its excellent optical and electronic properties. The optical band gap energy of CuS depends on its crystalline phase and is in the range of 1.48–2.89 eV, which matches the energy of ultraviolet and visible light (4.1–1.6 eV, 300–800 nm) [1,2], meaning that CuS has a strong absorption ability for ultraviolet and visible light and can be widely used in many fields, such as for the photocatalytic degradation of organic pollutants, solar cells, optical filters, and superconductors [3,4,5]. In the photocatalytic degradation of organic pollutants, CuS is a Fenton-like catalyst, a type of catalyst that effectively decomposes a wide range of organic pollutants in the presence of hydrogen peroxide (H_2_O_2_) with light [4,5]. Like other Fenton-like catalysts, CuS catalyzes the decomposition of H_2_O_2_ to generate a large number of hydroxyl radicals (^•^OH) and superoxide ions (^•^O_2_^−^) under light irradiation. Subsequently, ^•^OH and ^•^O_2_^−^ degrade the organic pollutants. The photocatalytic degradation of organic pollutants using CuS as a catalyst can be described by the following equations [4,5,6]:

CuS + light irradiation → h^+^ (CuS) + e^−^ (CuS) (1)

h^+^ (CuS) + H_2_O_2_ → ^•^OOH + H^+^ (2)

e^−^ (CuS) + H_2_O_2_ → ^•^OH + OH^−^ (3)

^•^OOH → ^•^O_2_^−^ + H^+^ (4)

Organic pollutants + ^•^OH + ^•^O_2_^−^ → degradation products (5)

where e^−^ (CuS) and h^+^ (CuS) are the electrons jumping from the valence band to the conduction band of CuS and the positively charged holes in the valence band of CuS, respectively. The symbol ^•^OOH refers to the hydroperoxy radical.

A large amount of research has suggested that the size reduction of CuS into the nanoscale dimension leads to significant changes in its physicochemical properties due to the quantum size effect [7,8,9]. Therefore, in the last few years, various techniques, such as the hydrothermal method, microwave irradiation, and electrodeposition have been developed to prepare different shapes for these CuS nanoparticles like nanospheres, nanoplates, nanotubes, nanorods, and flower-like structures [5,7,8,9,10,11,12]. Among these techniques, the hydrothermal method is the most commonly used one in the synthesis of CuS nanoparticles. For example, Cheng et al. introduced a hydrothermal method (reaction temperature, 120–180 °C; reaction time, 6–24 h) to synthesize ball-flower shaped CuS nanoparticles by mixing the solutions of copper chloride and thiourea using poly(vinylpyrrolidone) (PVP) as the surfactant. The morphologies of ball-flower shaped CuS are highly dependent on the concentration ratios of copper chloride to thiourea, the reaction temperature, and reaction time. Finally, this ball-flower shaped CuS shows high photocatalytic activity for the degradation of rhodamine B (RhB) under exposure to ultraviolet light irradiation [9]. Mezgebe and coworkers suggested a hydrothermal method to prepare nano-sized CuS by heating the solution containing Cu(NO_3_)_2_ and thiourea at 150 °C for 6 h. The different morphological structures of CuS were prepared by changing its hydrothermal solvents. The prepared nano-sized CuS showed high catalytic activity for the degradation of its model pollutant, methylene blue [11]. Generally, hydrothermal solvents have a significant effect on the morphology and activity of nano-sized CuS [5,11] and thus the development of versatile solvents is a research hotspot. In this context, the use of new type of solvent, ionic liquids (ILs), to hydrothermally produce nano-sized CuS has aroused great interest among researchers [13,14,15,16] because ILs have some unique properties, such as high viscosity and good solubility for a wide range of inorganic and organic compounds and their physicochemical properties can be easily adjusted by changing the chemical structures of their anions and cations [17,18]. Yao et al. reported the solvothermal synthesis of CuS nanowalls in the presence of a common IL, 1-dedyl-3-methylimidazolium bromide ([C_10_mim]Br) (heating at 150 °C for 10 h). This IL may be involved in the formation of the thiourea-Cu(II) complex via surface absorption or as one of elements in the complex, which can effectively modulate the morphology of CuS. Furthermore, the chain length of the IL cation, the IL anion’s nature, and the IL imidazolium ring also played an important role in delicately constructing the CuS nanowalls [13].

From the above discussion, there is no doubt that hydrothermal method with ILs as solvents is an ideal technique for the preparation of nano-sized CuS. However, this method usually operates at higher temperature, takes a longer time and does not maximize the effectiveness of ILs because the ILs used in the reported work only act as additives, which cannot reflect the advantages of ILs (such as their excellent solubility and higher viscosity). To make full use of the advantages of ILs, this work suggests a biphasic system composed of a hydrophobic IL phase that contains copper ions and a water phase that contains sulfide ions. The CuS nanoparticles would be generated in the interfaces between the two phases. The solubility of copper ions in the IL phase suggests the slow release of copper ions in the phase interfaces, and the high viscosity of the ILs suggests the slow diffusion of nanoparticles in the IL phase, which may facilitate the orderly growth of nanoparticles. Based on this conception, this work designed a biphasic system consisting of hydrophobic IL and water phases to produce CuS nanoparticles and investigated their photocatalytic activities.

## 2. Results and Discussion

In this work, CuS nanoparticles were prepared using a simpler route which was based on a two-phase system composed of hydrophobic ILs (1-octyl-3-methylimidazolium bis(trifluoromethylsulfonyl)imide ([C_8_mim]NTf_2_) and 1-butyl-3-carboxymethylimidazolium bis(trifluoromethylsulfonyl)imide ([C_4_C_2_OOHim]NTf_2_)) and water with copper(II) acetate (Cu(Ac)_2_) as a copper source and sodium sulfide (Na_2_S) and thioacetamide (TAA) as sulfur sources. Compared with the hydrothermal method (usually operating at 120–180 °C [9,11,13]), a traditional technique to produce CuS nanoparticles, the developed hydrophobic IL/water systems were actuated at mild conditions (the IL/Na_2_S systems were carried out at room temperature, and the IL/TAA systems were conducted at 80 °C). The results discussed below indicate that the prepared CuS nanostructures have a pronounced absorption ability for ultraviolet and visible light and exhibit good photocatalytic efficiency toward the degradation of the virulent organic pollutant, RhB.

### 2.1. Morphology and Structural Characterization of CuS

**Figure 1:**
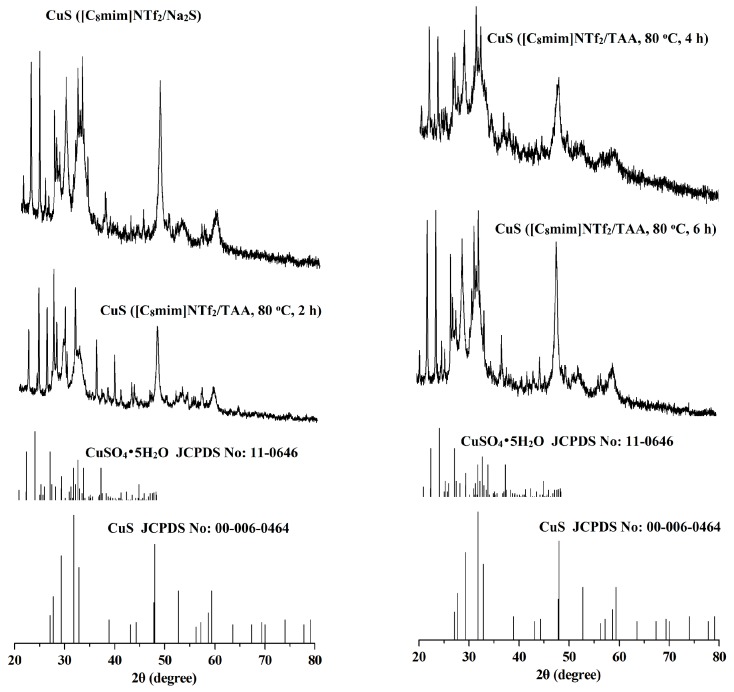
X-ray diffraction (XRD) patterns of the copper sulfide (CuS) prepared in the [C_8_mim]NTf_2_/water systems.

**Figure 2:**
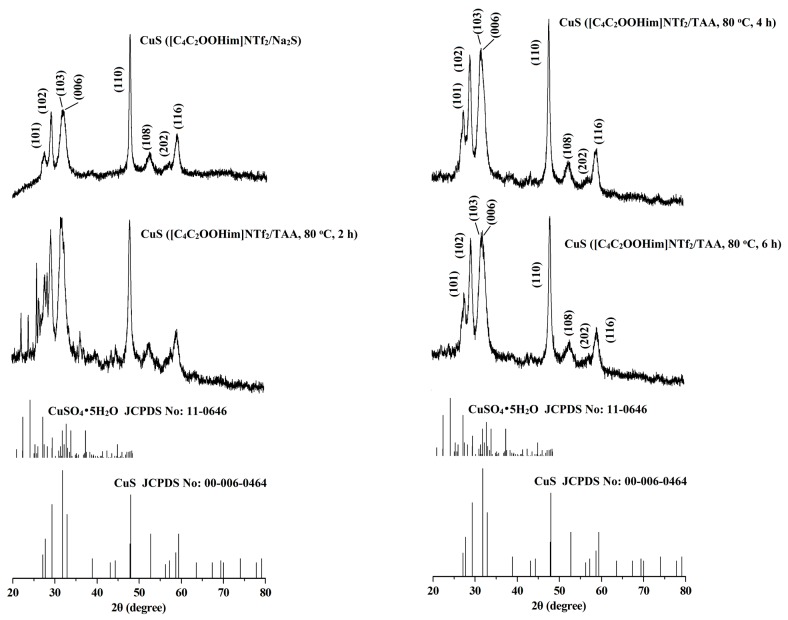
XRD patterns of the CuS prepared in the [C_4_C_2_OOHim]NTf_2_/water systems.

The crystal structures and phase purity of the CuS prepared by different systems were investigated by X-ray diffraction (XRD). The results are shown in Figure 1 and Figure 2. As can be seen from the two figures, for the CuS nanoparticles prepared from the interfaces of the [C_8_mim]NTf_2_/water systems and the [C_4_C_2_OOHim]NTf_2_/TAA system (80 °C, 2 h), there are some impurity peaks in the XRD spectra, which are the characteristic signals of CuSO_4_•5H_2_O. Meanwhile, a higher purity of CuS can be obtained when the [C_4_C_2_OOHim]NTf_2_/Na_2_S system is used as the reaction medium, which may ascribed to its acidic nature and strong complexation ability of the carboxyl group in the [C_4_C_2_OOHim]NTf_2_ with Cu^2+^_._ As shown in Figure 2, for the [C_4_C_2_OOHim]NTf_2_/TAA system, the heating time is also a key factor that affects the product’s purity. A shorter heating time (2 h) leads to small amounts of impurities in the product, and there are no impurity peaks found in the XRD spectra when the heating times were set at 4 h and 6 h.

**Figure 3:**
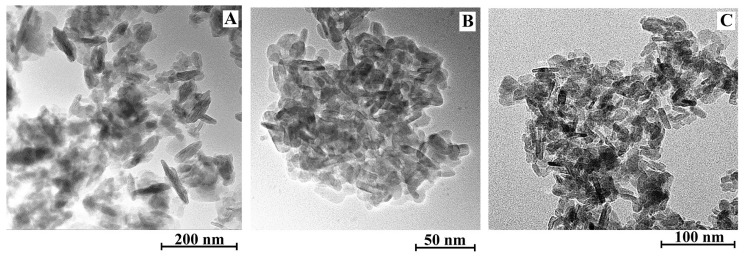
Transmission electron microscopic (TEM) images of the CuS prepared in different systems. (**A**) [C_4_C_2_OOHim]NTf_2_/Na_2_S, (**B**) [C_4_C_2_OOHim]NTf_2_/TAA (80 °C, 4 h) and (**C**) [C_4_C_2_OOHim]NTf_2_/TAA (80 °C, 6 h).

**Figure 4:**
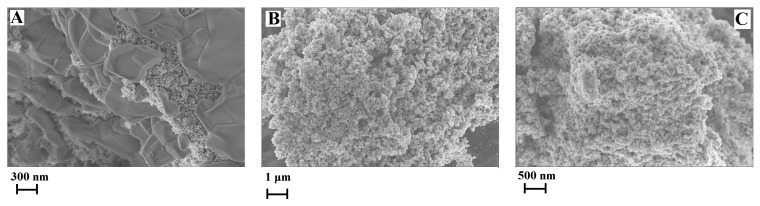
Field-emission scanning electron microscopic (FE-SEM) images of the CuS prepared in different systems. (**A**) [C_4_C_2_OOHim]NTf_2_/Na_2_S, (**B**) [C_4_C_2_OOHim]NTf_2_/TAA (80 °C, 4 h) and (**C**) [C_4_C_2_OOHim]NTf_2_/TAA (80 °C, 6 h).

Since the [C_4_C_2_OOHim]NTf_2_/Na_2_S system and the [C_4_C_2_OOHim]NTf_2_/TAA system (80 °C, 4 h or 6 h) can provide high purity products, they were selected for the following studies. The transmission electron microscopic (TEM) and field-emission scanning electron microscopic (FE-SEM) images of CuS obtained from the [C_4_C_2_OOHim]NTf_2_/Na_2_S and [C_4_C_2_OOHim]NTf_2_/TAA systems are shown in Figure 3 and Figure 4. As can be seen from Figure 3, the TEM images of the as-prepared CuS clearly show the formation of plate-like nanostructures and the particles tend to agglomerate to some extent. The SEM images (Figure 4) of CuS indicate that the CuS nanoparticles obtained from the [C_4_C_2_OOHim]NTf_2_/Na_2_S system self-assembled to form large plate structures and the CuS nanoparticles generated from [C_4_C_2_OOHim]NTf_2_/TAA systems are prone to form rough and spheroidic structures.

Additionally, the specific surface areas of the as-prepared CuS nanoparticles were analyzed by the Brunauer-Emmett-Teller (BET) method. It was found that the specific surface areas of the prepared CuS nanoparticles are 45.4 m^2^ g^−1^ ([C_4_C_2_OOHim]NTf_2_/Na_2_S system), 28.7 m^2^ g^−1^ ([C_4_C_2_OOHim]NTf_2_/TAA system, 80 °C, 4 h), and 18.3 m^2^ g^−1^ ([C_4_C_2_OOHim]NTf_2_/TAA system, 80 °C, 6 h), respectively, which are larger than those of the hydrothermally prepared CuS nanoplates [11].

### 2.2. Ultraviolet–Visible (UV/Vis) Spectra of CuS

**Figure 5:**
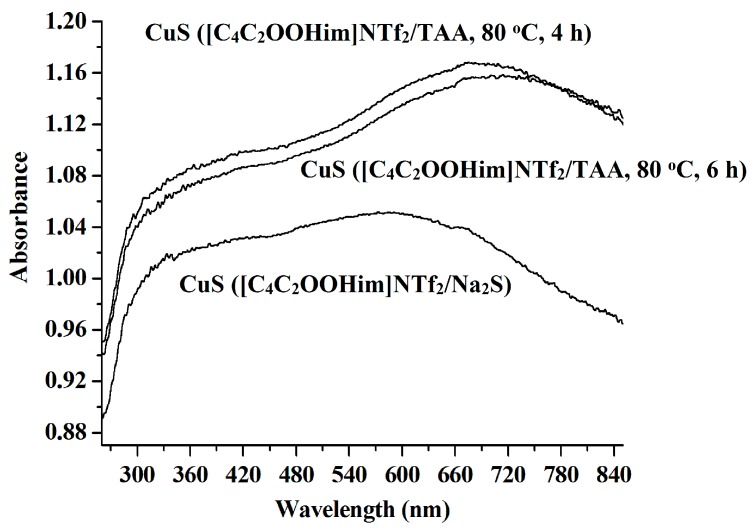
UV/Vis spectra of the CuS nanoparticles prepared in different systems.

The UV/Vis absorption spectra of the resulting CuS products are shown in Figure 5. It is clear that all the spectra show strong absorption feature in the UV/Vis region, which suggests the potential application of CuS in the fields of solar cells and photocatalysts. Moreover, the absorption peak of the CuS prepared from the [C_4_C_2_OOHim]NTf_2_/Na_2_S system (maximum absorption wavelength is around 582 nm) show a blue shift compared with the absorption peaks of the CuS produced from the [C_4_C_2_OOHim]NTf_2_/TAA system (maximum absorption wavelengths are about 679 nm), implying that the CuS nanoplates synthesized from the [C_4_C_2_OOHim]NTf_2_/Na_2_S system are more uniform in size and morphology (Figure 4) [19].

### 2.3. Catalytic Activities

**Figure 6:**
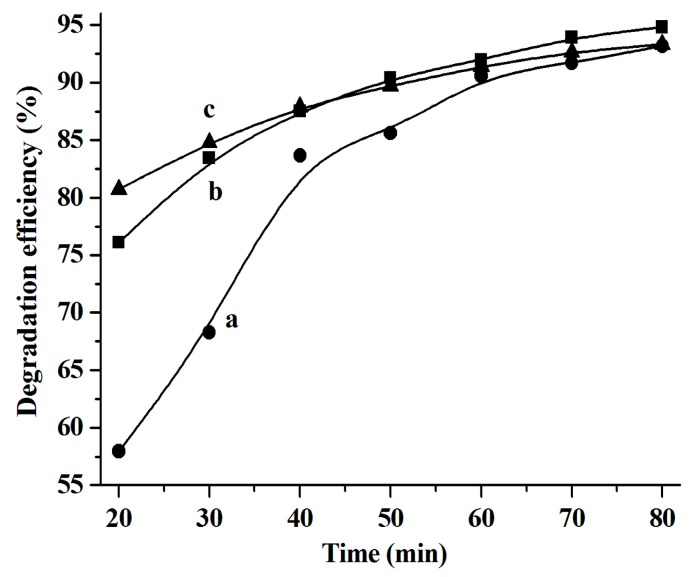
The rhodamine B (RhB) degradation efficiency catalyzed by the CuS produced from different systems. (a) [C_4_C_2_OOHim]NTf_2_/TAA (80 °C, 4 h), (b) [C_4_C_2_OOHim]NTf_2_/Na_2_S, and (c) [C_4_C_2_OOHim]NTf_2_/TAA (80 °C, 6 h); 5 mg of CuS, 25 mL of RhB (10 mg L^−1^), and 1.0 mL of H_2_O_2_ (30 wt.%).

**Figure 7:**
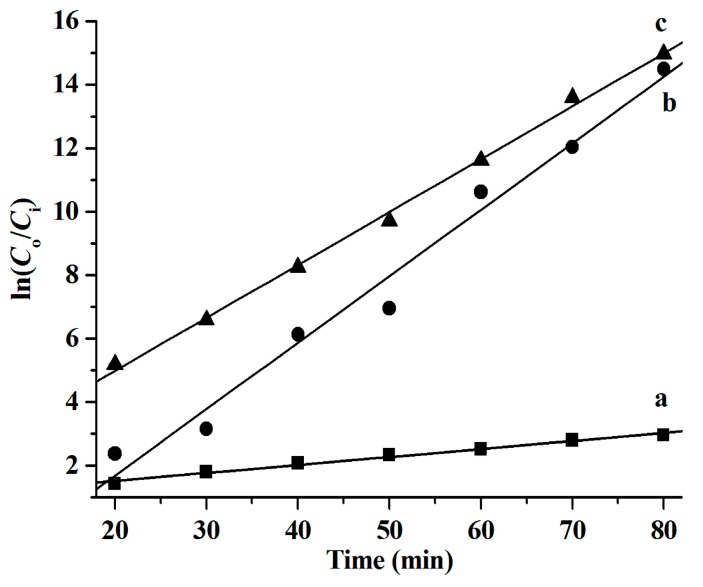
Degradation kinetics of RhB catalyzed by the CuS synthesized from different systems. (a) [C_4_C_2_OOHim]NTf_2_/Na_2_S, (b) [C_4_C_2_OOHim]NTf_2_/TAA (80 °C, 4 h), and (c) [C_4_C_2_OOHim]NTf_2_/TAA (80 °C, 6 h); 5 mg of CuS, 25 mL of RhB (10 mg L^−1^), and 1.0 mL of H_2_O_2_ (30 wt.%).

It has been reported that CuS nanoparticles are good photocatalysts for the degradation of organic dyes, such as RhB [6,9,20,21,22,23]. Herein, the photocatalytic performance of the as-synthesized CuS nanostructures for the degradation of RhB in the presence of H_2_O_2_ under natural light irradiation at ambient temperature was investigated. The results shown in Figure 6 illustrate that all the as-synthesized CuS nanoparticles exhibit high photocatalytic activity and over 90% of the RhB is decomposed after 60 min. To further characterize the catalytic performance of the prepared CuS, the relationship between ln (*C*_o_/*C*) and degradation time (min) was studied and the results shown in Figure 7 suggest that the RhB degradation conforms to the first-order kinetic model, which is in good agreement with the observations reported in the literature [6,21,24]. The RhB degradation kinetics can be expressed by the following equation:

ln(*C*_o_/*C_i_*) = *kt* (6)

where *k* refers to the reaction rate constant and *C*_0_ and *C_i_* are the initial and instant RhB concentrations, respectively.

It was found that the reaction rate constants (*k* values) of the RhB degradation catalyzed by CuS are 0.025 min^−1^ ([C_4_C_2_OOHim]NTf_2_/Na_2_S system), 0.209 min^−1^ ([C_4_C_2_OOHim]NTf_2_/TAA system, 80 °C, 4 h), and 0.167 min^−1^ ([C_4_C_2_OOHim]NTf_2_/TAA system, 80 °C, 6 h), respectively.

**Figure 8:**
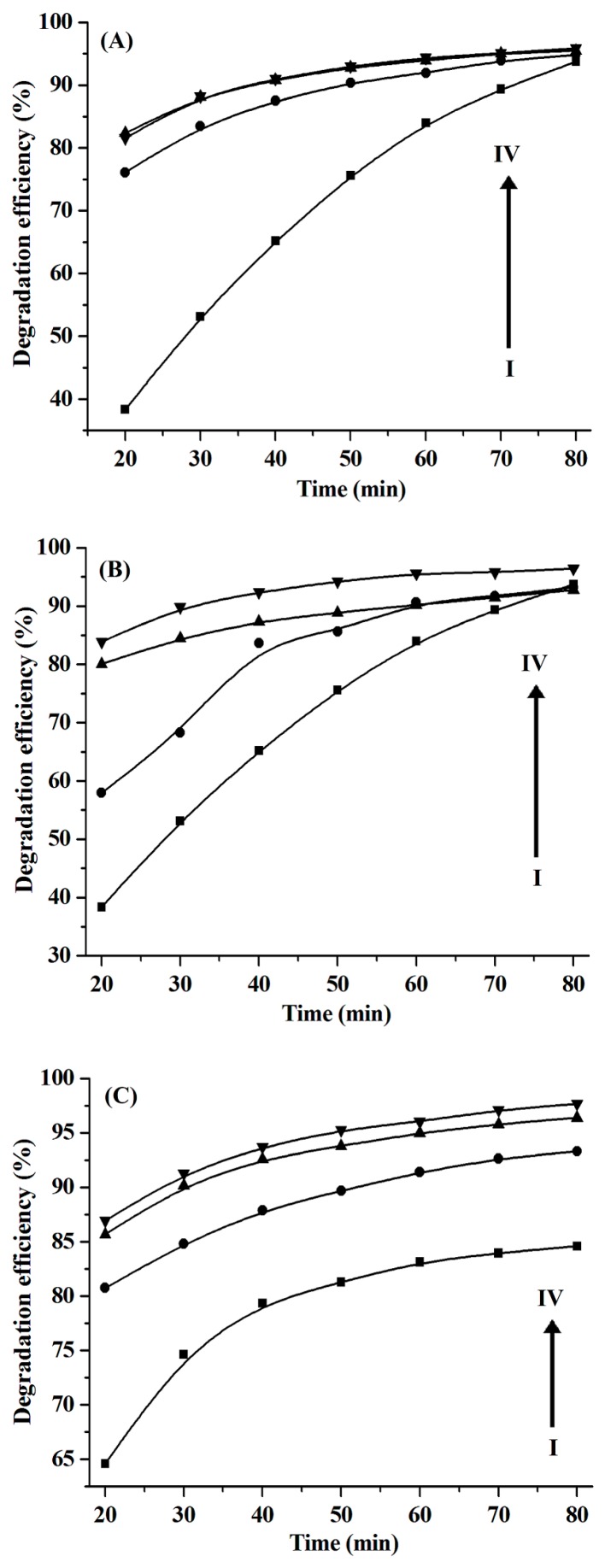
The influence of H_2_O_2_ dosage on the degradation efficiency of the RhB catalyzed by CuS prepared from different systems. (**A**) [C_4_C_2_OOHim]NTf_2_/Na_2_S system, (**B**) [C_4_C_2_OOHim]NTf_2_/TAA system (80 °C, 4 h) and (**C**) [C_4_C_2_OOHim]NTf_2_/TAA system (80 °C, 6 h); 5 mg of CuS, 25 mL of RhB (10 mg L^−1^); from I to IV, 0.59% of H_2_O_2_, 1.2% of H_2_O_2_, 1.7% of H_2_O_2_, and 2.2% of H_2_O_2_, respectively.

In addition, the dosage of H_2_O_2_ is one of key factors affecting the degradation efficiency of organic dyes [6,23]. Therefore, the influence of H_2_O_2_ dosage on the degradation efficiency was investigated, and the results shown in Figure 8A indicate that the degradation efficiency of the CuS nanoplates obtained from the [C_4_C_2_OOHim]NTf_2_/Na_2_S system increases as the H_2_O_2_ dosage increases from 0.59% (wt.%) to 1.7% and remains constant with an increasing H_2_O_2_ dosage up to 2.2%. That is to say, 1.7% of H_2_O_2_ is a better choice for the degradation of RhB by CuS nanoplates. As illustrated in Figure 8B, the degradation efficiency of the CuS nanospheres obtained from the [C_4_C_2_OOHim]NTf_2_/TAA system (80 °C, 4 h) also increases by increasing the H_2_O_2_ dosage from 0.59% to 2.2% and when 2.2% of H_2_O_2_ is adopted, the degradation efficiency of RhB increases up to 92.4% after 40 min of reaction. Thus, 2.2% is regarded as the optimal H_2_O_2_ dosage for the CuS nanopheres ([C_4_C_2_OOHim]NTf_2_/TAA, 80 °C, 4 h). For the CuS nanospheres produced from the [C_4_C_2_OOHim]NTf_2_/TAA system (80 °C, 6 h), their catalytic capacity also increases by increasing the H_2_O_2_ dosage from 0.59% to 2.2% (Figure 8C), and the degradation efficiency goes above 90% when 1.7% of H_2_O_2_ is used after 30 min. Thus, 1.7% is regarded as the optimal H_2_O_2_ dosage for the CuS nanopheres obtained from the [C_4_C_2_OOHim]NTf_2_/TAA system (80 °C, 6 h).

Recently, some excellent work has reported the use of CuS nanostructures with different morphologies to decompose RhB [6,9,20,21,23]. Therefore, a comparison of the photocatalytic performance between the CuS nanoparticles synthesized in the present work and those reported in the literature was conducted, and the results are listed in Table 1. As can be seen, the degradation of RhB by CuS hollow nanospheres [20], nanoneedles [21], nanospheres [23] and ball-flowers [9] was conducted under an extra light source, such as Xe and mercury lamps. However, the RhB degradation reaction catalyzed by the CuS synthesized in this work can be carried out under natural sunlight. This is an advantage of the as-prepared CuS nanoparticles. Furthermore, more than 90% of RhB was degraded after 30–40 min of reaction, suggesting that the as-prepared CuS has a stronger photocatalytic capacity.

Finally, it should be noted that CuS nanoparticles have good biocompatibility [25,26] and the ILs used in this work have very low volatility, whereby any air pollution caused by them can be significantly reduced [27]. Furthermore, after reaction, the ILs can be recycled through washing with HCl (0.2 mol L^−1^) and drying (60 °C for 4 h) processes. Therefore, the suggested IL/water systems are environmentally friendly methods for the preparation of CuS nanoparticles.

## 3. Materials and Methods

### 3.1. Materials

Copper(II) acetate monohydrate (Cu(Ac)_2_·H_2_O, 99%), thioacetamide (TAA, ≥99%), 1-methylimidazole (MI, 99%), rhodamine B (RhB, 99%), and hydrogen peroxide solution (H_2_O_2_, 30 wt.% in water) were obtained from Aladdin Chemical Co., Ltd. (Shanghai, China). The ILs, 1-octyl-3-methylimidazolium bis(trifluoromethylsulfonyl)imide ([C_8_mim]NTf_2_, 99%) and 1-butyl-3-carboxymethylimidazolium bis(trifluoromethylsulfonyl)imide ([C_4_C_2_OOHim]NTf_2_, 95%) were purchased from Chengjie Chemical Co., Ltd. (Shanghai, China). Unless otherwise stated, all other reagents used were of analytical grade. Ultrapure water produced by an Aquapro purification system (18.2 MΩ cm, Aquapro International Co., Ltd., Dover, DE, USA) was used throughout the experiments.

### 3.2. Preparation of Nano CuS

The preparation of the nano CuS in the [C_8_mim]NTf_2_ + MI/water system was as follows: 11 mL of [C_8_mim]NTf_2_ (containing 9.1% (volume percentage) of MI) and 20 mL of Cu(Ac)_2_ solution (0.1 mol L^−1^) were mixed under stirring at room temperature for 30 min. Here, MI acted as a complexant, i.e., it could form complexes with copper ions, resulting in the transfer of copper ions to the IL phase. The water phase was discarded and the IL phase was washed with water to remove the undissolved Cu(Ac)_2_, and then Na_2_S (22 mL, 0.1 mol L^−1^) was added dropwise to the IL phase under stirring for 15 min. The color of the IL phase changed from blue to black, indicating the formation of CuS. The IL phase was dissolved with ethanol to precipitate CuS. The products were washed several times with ethanol, followed by washing with water and acetone, the resultant CuS was dried at 40 °C for 2 h.

When TAA was used as a sulfur source, the preparation of CuS followed a similar pattern, except that TAA (0.1 mol L^−1^, prepared with 0.12 mol L^−1^ of HCl solution) was used instead of Na_2_S, and the reaction was conducted at 80 °C [28].

Preparation of the nano CuS in [C_4_C_2_OOHim]NTf_2_ followed a similar procedure (as described above), except [C_4_C_2_OOHim]NTf_2_ (without the addition of MA or HCl) was used instead of [C_8_mim]NTf_2_.

All CuS samples were characterized by an X-ray diffractometer (XRD, model X’Pert PRO MPD, PANalytical B.V., Almelo, Netherlands), a field-emission scanning electron microscope (FE-SEM, Quanta 250 FEG, Thermo Fisher Scientific, Hillsboro, OR, USA), a transmission electron microscope (TEM, model Tecnai G2 20, FEI, Hillsboro, OR, USA), a surface area and porosity analyzer (model ASAP 2460, Micromeritics Instrument Corp., Norcross, GA, USA) and an ultraviolet-visible/near infrared spectrophotometer (Model UH4150, Hitachi High-Technologies Corp., Tokyo, Japan).

### 3.3. Measurement of Photocatalytic Activity

The photocatalytic activity of the prepared CuS nanoparticles was evaluated by degrading the model compound, RhB, in aqueous media under natural sunlight irradiation. The experiments were conducted according to the procedures reported in the literature [6,9,20,21]. Typically, 5.0 mg of CuS nanoparticles was added in the RhB solution (25 mL, 10 mg L^−1^) and the resultant reaction mixture was magnetically stirred in the dark for 30 min to obtain adsorption–desorption equilibrium. After that, a certain amount of H_2_O_2_ (30 wt.%) was added into the reaction mixture, and the photocatalytic degradation of RhB was initiated by natural light irradiation. The RhB concentration in the reaction mixture was measured by an ultraviolet-visible (UV/Vis) spectrophotometer (model TU-1810, Purkinje General Instrument Co., Beijing, China) at 550 nm. The degradation efficiency was calculated by using the following equation:

Degradation efficiency = (1 − *C*_i_/*C*_o_) × 100% (7)

where *C*_o_ and *C*_i_ are the initial and instant RhB concentrations, respectively.

## 4. Conclusions

The present work has suggested a facile method to prepare CuS nanoparticles from the interfaces of hydrophobic IL and water phases. The morphologies of CuS could be selectively produced by changing the types of their sulfur sources. The use of Na_2_S led to the production of CuS nanoplates and CuS nanospheres could be obtained when TAA is used as a sulfur source. All the prepared CuS nanostructures exhibited strong UV/Vis absorption and excellent photocatalytic activities toward the degradation of RhB under exposure to natural sunlight irradiation. Compared with the reported techniques, the suggested two-phase method exhibited some advantages for the preparation of CuS, such as mild reaction conditions and simplified reaction procedures. This facile and eco-friendly strategy is expected to produce other metal sulfide nano-structures with great promise for various applications.

## Figures and Tables

**Table 1:** Comparison of the photocatalytic performance of CuS nanoparticles prepared by this work with reported CuS nanostructures.

Catalyst	Mass Ratio of RhB to Catalyst (*m*_RhB_/*m*_CuS_)	Degradation Time (min)	Irradiation Source	Degradation Efficiency (%, *v*/*v*)	Reference
Flower-like CuS hollow nanospheres	10^−2^	40	Xe lamp (150 W)	>90%	20
CuS nanoneedles	1.25 × 10^−2^	105	Mercury lamp (500 W)	91%	21
CuS hierarchical microflowers	6.0 × 10^−2^	55	Natural light	95%	6
CuS nanospheres	2.0 × 10^−2^	20	Xe lamp (300 W)	About 100%	23
CuS ball-flowers	4.8 × 10^−2^	60	Mercury lamp (300 W)	About 100%	9
CuS nanoplates	5.0 × 10^−2^	40	Natural light	90.8%	This work
CuS nanospheres	5.0 × 10^−2^	30	Natural light	90.2%	This work
